# Unaltered empathy-related behaviors in Williams–Beuren syndrome mouse models

**DOI:** 10.1186/s13041-026-01278-2

**Published:** 2026-03-11

**Authors:** Dahm So, Hye Lim Cha, Sua Lee, Sowon Kim, Eunsu Yoo, Sehoon Keum

**Affiliations:** 1 Center for Memory and Glioscience, Institute for Basic Science, Daejeon, 34126 South Korea; 2 Department of Bio and Brain Engineering, Korea Advanced Institute of Science and Technology, Daejeon, 34141 South Korea; 3 Department of Biomedical Sciences, Seoul National University, Seoul, 03080 South Korea

**Keywords:** Williams–Beuren Syndrome, Heightened empathy, Observational fear, Affiliative allogrooming

## Abstract

Williams–Beuren Syndrome (WBS), a neurodevelopmental disorder caused by a heterozygous microdeletion at chromosome 7q11.23, is characterized by hypersociability and enhanced affective empathy. However, the specific genetic and neural mechanisms within the WBS locus underlying this elevated empathic response remain unknown. Here, we investigated empathy-related behaviors, including observational fear and allogrooming, in WBS mouse models harboring a deletion within the conserved syntenic region on mouse chromosome 5. We demonstrate that WBS mice exhibited emotional contagion and prosocial consolation behaviors comparable to their wild-type controls. Furthermore, WBS mice with single-gene deletions of the cortex-enriched genes *Abhd11*, *Limk1*, *Mlxipl*, and *Stx1a* also showed unaffected empathic freezing behavior. Collectively, our findings suggest that the enhanced empathic responsiveness reported in individuals with WBS may be influenced by reduced social inhibition toward others, while acknowledging that limitations of current rodent behavioral assays preclude definitive conclusions regarding primary neural mechanisms of empathy.

**Supplementary Information:**

The online version contains supplementary material available at 10.1186/s13041-026-01278-2.

## Main text

**Fig. 1:**
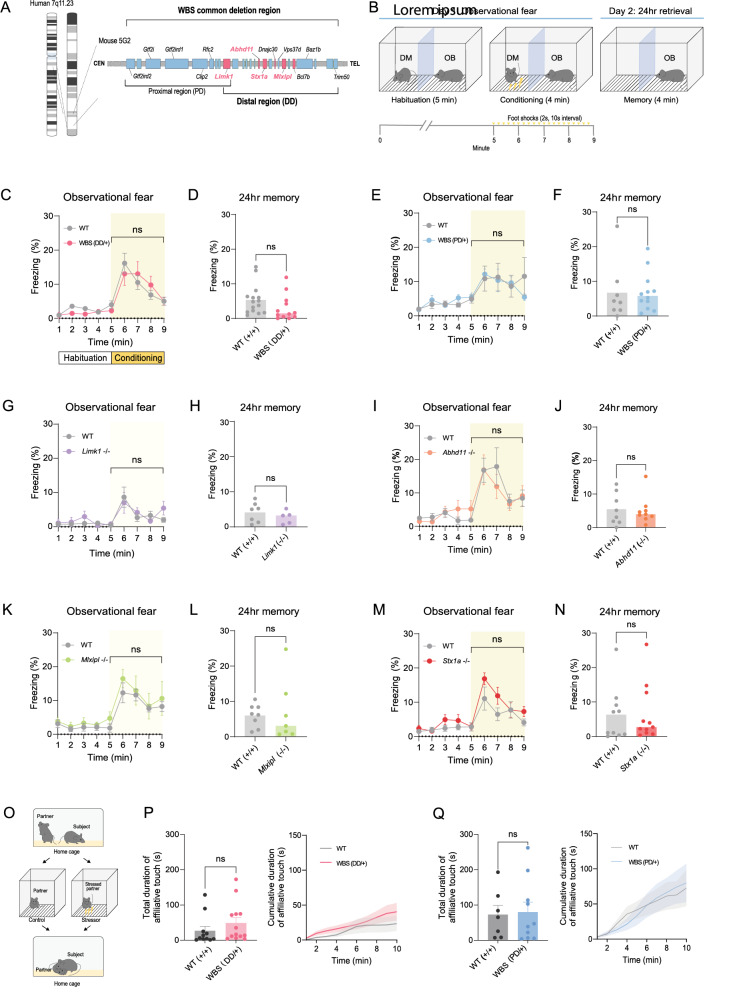
Unaltered observational fear and allogrooming behaviors in WBS mice.** A** Comparative schematic of the 7q11.23 chromosomal region in humans and its syntenic region on mouse chromosome 5, highlighting the WBS locus. Cortex-enriched genes (*Abhd11*, *Mlxipl*, *Limk1*, and *Stx1a*) are indicated in red. **B** Diagram of the observational fear chamber and outline of the behavioral paradigm. **C, D** WBS (DD/+) mice (*n* = 12) showed no difference in observational fear (C) or 24-h retrieval (D) compared to wild-type (WT) littermates (*n* = 15). **E, F** Vicarious freezing (E) and 24-h contextual memory (F) were similar in WBS (PD/+) mice (*n* = 12) and WT controls (*n* = 9). **G, H** *Limk1* knockout (KO) mice (*n* = 5) exhibited observational fear (G) and 24-h retrieval (H) similar to WT mice (*n* = 7). **I, J** Vicarious freezing (I) and 24-h contextual memory (J) were comparable between *Abhd11* KO mice (*n* = 9) and WT controls (*n* = 8). **K, L** *Mlxipl* KO mice showed no difference in vicarious freezing (*n* = 11) or 24-h memory (*n* = 6) compared to WT littermates (*n* = 8). **M, N** No difference in observational fear (M) or 24-h retrieval (N) between *Stx1a* KO mice (*n* = 11) and their WT mice (*n* = 10). **O** Schematic of the prosocial interaction between a subject and a partner. Control group partners were separated from subjects but received no foot shocks during separation. **P, Q** Total and cumulative duration of affiliative allogrooming behavior toward a stressed conspecific in WBS distal deletion (DD/+) mice (WT, *n* = 11; DD/+ , *n* = 13) and WBS proximal deletion (PD/+) mice (WT, *n* = 7; PD/+ , *n* = 10), compared with their respective WT littermate controls. *p < 0.05, **p < 0.01. ns, not significant. Unpaired t-tests were used for behavioral data in panels C–F, I, J, M, and N; Mann–Whitney U tests were used for panels G, H, K, and L; and Kruskal–Wallis tests were used for panel P. Data are presented as mean ± SEM

Empathy, the ability to recognize, understand, and share others’ affective states, is fundamental for social interaction and mental well-being [1]. Despite its significant heritability and dysregulation in various neuropsychiatric conditions, the specific genetic determinants of empathic abilities remain largely elusive. This gap highlights the importance of Williams–Beuren Syndrome (WBS), a neurodevelopmental disorder caused by haploinsufficiency of approximately 26 genes within the WBS critical region on chromosome 7q11.23 (Fig. 1A). Individuals with WBS are characterized by pronounced hypersociability, including reduced social inhibition and an increased tendency to approach unfamiliar others. In parallel, WBS has been associated with reportedly enhanced empathic responsiveness, particularly at the level of affect sharing, such as heightened emotional reactivity to others’ emotional states [2]. Consistent with this distinction, WBS is associated with atypical neuroanatomy, including altered activation and connectivity between prefrontal cortical regions and the amygdala—key nodes for social–emotional processing [3]. However, despite the prominent behavioral phenotype, the precise mechanisms by which specific genes within the WBS locus contribute to the heightened empathic response in WBS individuals are still unexplored.

To investigate whether haploinsufficiency of genes within the WBS critical region alters empathy-related behaviors, we utilized an observational fear task [4]. In this paradigm, an observer mouse develops context-dependent fear vicariously without directly receiving aversive stimuli while witnessing a demonstrator mouse receive repetitive foot shocks (Fig. 1B). This vicarious freezing behavior is a form of emotional contagion, considered a basic feature of affective empathy in mice [1, 4]. We measured vicarious freezing behaviors in observer mice carrying heterozygous microdeletions spanning distinct segments within the WBS region [5]. These models included mice with either a proximal deletion (PD/+, spanning *Limk1*–*Gtf2i*) or a distal deletion (DD/+, spanning *Trim50*–*Limk1*) (Fig. 1A). We found that mice harboring the heterozygous distal deletion (DD/+) exhibited observational fear responses comparable to their wild-type (WT) littermates (Fig. 1C). Furthermore, 24-h memory recall for the conditioned context was also unaffected in DD/+ mice (Fig. 1D). Similarly, mice with the heterozygous proximal deletion (PD/+) displayed vicarious freezing behavior and 24-h memory recall that did not significantly differ from WT controls (Fig. 1E, F). Collectively, these data suggest that heterozygous deletion of either the proximal or distal segment of the WBS critical region does not significantly alter empathic freezing behavior.

Recognizing that heterozygous deletions in mouse models of human haploinsufficiency syndromes may not always manifest clear phenotypes, possibly due to genetic background or incomplete penetrance [6], we subsequently investigated individual gene functions using homozygous null mutations. While homozygous null mutations do not perfectly replicate WBS’s hemizygous gene dosage, this approach allowed us to uncover potentially stronger or more subtle phenotypes and facilitated mechanistic studies, particularly when heterozygous effects were absent. Previous human brain transcriptomics analysis revealed distinct spatiotemporal expression patterns for the 28 WBS genes, notably highlighting *Abhd11*, *Limk1*, *Mlxipl*, and *Stx1a* in the distal WBS region as potential contributors to WBS phenotypes through their role in cortical neuron differentiation [7]. Given the pivotal role of prefrontal anterior cingulate cortex (ACC) in empathic ability [1, 4, 8], we examined how the complete loss-of-function of these cortex-enriched individual genes affects observational fear (Fig. 1A). First, we generated a knockout (KO) allele of the *Limk1* gene (Supplementary Fig. 1^1^A–C) and tested its effect on vicarious freezing behavior. *Limk1* KO (*Limk1−*/*−*) mice showed vicarious freezing behavior similar to their WT littermates (Fig. 1G, H). Similarly, observer mice with homozygous single-gene deletions of the other three genes (*Abhd11−*/*−*, *Mlxipl−*/*−*, and *Stx1a−*/*−*) also exhibited observational fear and 24-h memory recall that did not significantly differ from their respective WT controls (Fig. 1I–N). Next, we assessed affiliative social touch behavior in WBS (DD/+) and WBS (PD/+) mice when presented with conspecifics that had experienced electric shocks (Fig. 1O). This affiliative touch behavior, which was defined as the combination of allogrooming and body contact, represents a prosocial behavior aimed at providing comfort to distressed partners [9]. These responses were similar to those of their WT littermates in both WBS (DD/+) and WBS (PD/+) mice (Fig. 1P, Q). These findings indicate that WBS (DD/+) and WBS (PD/+) mice showed no significantly different motivation to provide comfort through affiliative touch [9].

In conclusion, the present study demonstrates that WBS mouse models do not exhibit increased empathic freezing behavior. Previous studies have shown that proximal deletions (PD/+) are associated with elevated sociability and altered fear responses, whereas distal deletions (DD/+) are more closely linked to cognitive deficits [5]. Specifically, the *Gtf2i* gene within the PD region is linked to social interactions, with its heterozygous deletion leading to increased sociability in association with myelination [10]. Furthermore, single-gene studies have demonstrated that *Limk1* KO mice have abnormal spine morphology, altered synaptic function, enhanced cued fear responses, and impaired spatial learning [11], and *Stx1a* KO mice display fear memory deficits and elevated social investigation behavior [12]. Despite these established neurophysiological and social phenotypes, we found that observational fear–dependent emotional contagion was preserved across both multi-gene heterozygous deletion models (PD/+ and DD/+) and individual gene KO WBS mouse lines. Similarly, affiliative allogrooming behavior was not altered in either PD/+ or DD/+ mice, further supporting the notion that core components of affective empathy may be preserved in these models. While a complete deletion model may more closely reflect the canonical human WBS genotype, partial deletion models remain informative for dissecting gene-specific contributions within the WBS locus [3, 13]. Nonetheless, we cannot exclude the possibility that combined dosage effects across the entire WBS region may be required to produce detectable alterations in empathy-related behaviors.

Given that hypersociability is a unique and intriguing feature of individuals with WBS [2], our results suggest that their increased empathic responsiveness might be a secondary consequence of reduced social inhibition toward others, rather than a primary alteration in neural mechanisms of empathy. This finding aligns with human studies demonstrating that individuals with WBS exhibit difficulties with higher-order social-cognitive functions while showing less severe impairment in basic perception-based affect sharing systems [2, 14]. An alternative perspective posits that, despite the conserved fundamental neurobiological basis of empathy between rodents and humans [4, 15], observational fear may not fully encompass the intricate dimensions of human affective empathy. Given the multifaceted nature of human empathy, which includes emotional arousal, awareness, understanding, and regulation [1, 15], the augmented empathic response in WBS might arise from differences in other affective capacities, such as emotion recognition. Therefore, future studies exploring these distinct components within relevant animal models are essential for advancing our understanding of the neural mechanisms underlying social-affective empathic capacities.

## Supplementary Information

Additional file 1

Additional file 2

## Data Availability

All data and materials are available from the corresponding author upon request.

## Abbreviations

WBS: Williams–Beuren Syndrome
OB: Observer
DM: Demonstrator
DD: Distal deletion
PD: Proximal deletion
WT: Wild type
KO: Knockout
ACC: Anterior cingulate cortex
