# Supplementary material for: Unaltered empathy-related behaviors in Williams–Beuren syndrome mouse models
Source: Mol Brain. 2026 Mar 11;19:27. doi: 10.1186/s13041-026-01278-2 (PMC13094111; doi:10.1186/s13041-026-01278-2)
Supplement: Supplementary file 1 — Additional file 1 [file 13041_2026_1278_MOESM1_ESM.docx]

**Methods**

Mice

Inbred strain C57BL/6J (B6J; JAX #000664), Del(5*Limk1*-*Trim50*)2Uta/J (WBS (DD/+); JAX #023888), Del(5*Gtf2i*-*Limk1*)1Uta/J (WBS (PD/+); JAX #023885), B6.129S6-*Mlxipl*^tm1Kuy^/J (JAX #010537), and *Stx1a*^tm1.1Sud^/J (JAX #008137) mice were obtained from The Jackson Laboratory and bred in-house for experiments. C57BL/6N-*Abhd11*^em1(IMPC)Mbp^/Mmucd (MMRRC #046419-UCD) strain was obtained from the MMRRC. Before behavioral experiments, mouse strains on a mixed genetic background were backcrossed for at least five generations to the wild-type C57BL/6J strain. Male mice aged 5-13 weeks were used for behavioral experiments. All experiments were performed between 12-6pm during the light cycle.

**Behavior tests**

Mice were transferred in their home cage to the test room at least 30 minutes before the test. For each behavior test, naive mice were used that had never been tested.

**Observational fear conditioning**

The observer and demonstrator mice were placed on each side of a chamber separated by a transparent partition perforated with holes to allow airflow. Following a 5-min habituation period, the demonstrator received twenty foot shocks (1 mA, 2 s duration, 10 s inter-shock interval). One day later, the observer mouse was returned to the chamber alone to assess contextual memory. Freezing behavior was quantified during the observational fear session (9 min total, including habituation and conditioning) and during the contextual memory test (4 min), and was defined as immobility lasting longer than 0.75 s, automatically scored using FreezeFrame software (ACTIMETRICS). To ensure adequate statistical power, we conducted an a priori power analysis based on observational freezing data obtained from more than 30 wild-type C57BL/6J mice [1, 2]. This analysis indicated a large effect size for the increase in freezing from habituation to conditioning (Cohen’s d ≈ 1), such that a sample size of 7–8 animals per genotype was sufficient to achieve 80% power at a significance level of α = 0.05.

**Allogrooming affiliative touch behavior**

Male pairs were housed together for more than 5 weeks. In each pair, one mouse was assigned as the subject and the other as the stressed partner. The stressed partner received 20 shocks at 0.75mA for 1 second, with random intervals of 20 or 40 seconds between shocks. During this time, the subject mouse stayed in the home cage inside a sound-proof chamber. After receiving shocks, the stressed partner returned to the home cage and freely interacted with the subject for 10 minutes. In the control condition, the subject was reunited with a partner that had been separated but had not received foot shocks. Allogrooming behavior was counted manually when the subject mouse held the stressed partner with its front paws and licked fur.

**Generation of *Limk1* knockout (KO) mice**

CRISPR/Cas9 technology was utilized to generate a null allele of the *Limk1* gene (NCBI Reference Sequence: Gene ID 16885). The *Limk1* KO mouse was designed by deleting 144 bp from exon 3 (ENSMUSE00001306814) and 232 bp from intron 3 within the *Limk1* gene (Supplementary Fig. 1). The 376 bp deletion was confirmed by Sanger DNA sequencing after cloning the PCR product from positive F0 generation *Limk1* mouse. A positive F0 mouse was bred with C57BL/6J mice for three generations before intercrossing heterozygous *Limk1*+/- mice to produce homozygous *Limk1* KO animals. Genotyping of the *Limk1* gene was performed using standard PCR conditions (94°C for 2 min; 30 cycles of 94°C for 30 s, 60°C for 30 s, 72°C for 30 s). The genotyping primer set (forward primer: 5’-agggaccgtacacacatgataa-3’; reverse primer: 5’-ctggcctggagctcactata-3’) amplifies a 376-bp PCR product from the KO allele and a 742-bp PCR product from the WT allele.

**Western blot**

Mice were anesthetized with isoflurane and decapitated for brain extraction. The brain sample was isolated on ice and acutely homogenized with ice-cold brain homogenization buffer (0.32M sucrose, 10mM HEPES, 2mM EDTA, 2mM EGTA, protease inhibitors, and phosphatase inhibitors, pH 7.4). Proteins were separated by electrophoresis on 10% Tris-Glycine gels and resolved proteins were transferred to nitrocellulose membranes. Membranes were incubated with LIMK1 (Abcam #ab217690, 1: 1,000) and GAPDH (Santa Cruz #sc-365062, 1:1,000) antibodies overnight at 4°C. HRP-conjugated secondary antibody signals were visualized using ChemiDoc XRS+ imaging system (BioRad, USA) and immunoblot signals were quantified using ImageJ software*.*

**Statistical analysis**

Statistical analyses were performed using GraphPad Prism 10, and all data are presented as mean ± standard error of the mean (SEM). Normality of data distributions was assessed using the Shapiro–Wilk test. For comparisons between two groups, unpaired two-tailed t-tests were applied when data met assumptions of normality; otherwise, the nonparametric Mann–Whitney U test was used. Results with p ≥ 0.05 are reported as not significant (ns), whereas * and ** denote p < 0.05 and p < 0.01, respectively. The statistical test used, exact sample sizes, and levels of statistical significance are specified in each figure legend.

**Reference**

1. Keum S, Kim A, Shin JJ, Kim JH, Park J, Shin HS. A Missense Variant at the Nrxn3 Locus Enhances Empathy Fear in the Mouse. Neuron 2018, 98(3):588-601 e585.

2. Keum S, Park J, Kim A, Park J, Kim KK, Jeong J, Shin HS. Variability in empathic fear response among 11 inbred strains of mice. Genes Brain Behav 2016, 15(2):231-242.
