# Supplementary material for: Unaltered empathy-related behaviors in Williams–Beuren syndrome mouse models
Source: Mol Brain. 2026 Mar 11;19:27. doi: 10.1186/s13041-026-01278-2 (PMC13094111; doi:10.1186/s13041-026-01278-2)
Supplement: Supplementary file 2 — Additional file 2 [file 13041_2026_1278_MOESM2_ESM.docx]

**Figure S1**. Generation of *Limk1* knockout mice. **A** Schematic illustration of the target site in exon 3 of the mouse *Limk1* locus. Exonic sequences are capitalized, intronic sequences are lowercase, and the single-guide RNA (sgRNA) target is underlined. DNA chromatograms for the target site in a wild-type (WT) mouse and a heterozygous founder mouse show a 376 bp deletion (144 bp from exon 3, 232 bp from intron 3), resulting in a frameshifted sequence. **B** PCR genotyping of *Limk1* knockout alleles. The WT band is 742 bp, and the knockout (KO) band is 376 bp. Lanes are labeled for wild-type (+/+), heterozygous (+/-), and homozygous knockout (-/-) mice. **C** Western blot analysis of brain lysates confirms the absence of LIMK1 protein in homozygous KO mice (-/-).
